# Enhancing Rural Children’s Cognitive Abilities Through Teacher Support: Quasi-Experimental Evidence from Longitudinal Data in China

**DOI:** 10.3390/jintelligence14010015

**Published:** 2026-01-16

**Authors:** Xinxin Hao, Jingxuan Lou, Mengyun Jin, Yihao Tian

**Affiliations:** 1 Institute for Advanced Study in Humanities and Social Sciences, Chengdu University, Chengdu 610106, China; haoxinxin@cdu.edu.cn; 2 School of Public Administration, Sichuan University, Chengdu 610065, China; loujingxuan0822@163.com (J.L.); jinmengyun@stu.scu.edu.cn (M.J.)

**Keywords:** rural education, teacher support, cognitive ability, developmental inequality

## Abstract

This study leverages longitudinal data from the China Family Panel Studies (CFPS, 2012–2020) to examine the association between teacher support and cognitive ability among children aged 10–16 living in economically disadvantaged rural areas of China. Employing a difference-in-differences (DID) framework, we found that exposure to the Rural Teacher Support Program (RTSP) is associated with an improvement of about 0.19 standard deviations in students’ cognitive abilities after accounting for individual-, family-, and county-level characteristics. Two key mechanisms appear to underlie this association, reflected in increased teacher quantity and enhanced student satisfaction with teachers. Heterogeneity analyses further show that these benefits are more pronounced among female students and those from low-income households, suggesting that teacher-centered institutional improvements may help mitigate developmental disparities. Overall, the longitudinal results indicate that better teacher-related environments are likely to support children’s cognitive development, which in turn may help reduce educational inequality in under-resourced areas.

## 1. Introduction

Cognitive ability is an individual’s intrinsic capacity to process, store, and retrieve information (Cattell, 1987). It is commonly understood to include both fluid intelligence and crystallized intelligence. Fluid intelligence reflects innate abilities in reasoning, problem-solving, and abstract thinking (Schubert et al., 2020). Crystallized intelligence reflects the knowledge individuals build up through schooling and life experience, such as vocabulary, reading, and numerical skills (Schubert et al., 2020). According to human capital theory, cognitive ability plays a central role in human capital, as it enhances individuals’ capacity to learn, acquire skills, and perform productively throughout life (Hanushek, 2009). Recent empirical work has demonstrated that cognitive competence not only affects educational achievements and income but is also related to other aspects of well-being, such as health and intergenerational mobility (Jokela, 2022; Ozawa et al., 2022; Marks, 2023). Empirical evidence also shows that people who perform better than their parents in cognitive ability tend to be more upwardly mobile (McGue et al., 2020).

Among the many influences on cognitive development, formal schooling has long been viewed as a particularly direct and context-sensitive pathway (Heckman & Kautz, 2013). Compared with home educational support, school is a more structured and policy-responsive environment. It can help narrow the gaps in human capital investment among children from different families with varying levels of income and parental education (W. Wu et al., 2023). In this institutional environment, teachers represent the most essential resource for promoting cognitive development. Teachers influence children’s cognitive outcomes through frequent and close interactions (Jia & Liu, 2017). The role of teachers is particularly pronounced in poor rural settings, where limited home-based support heightens the need for skilled educators who can offset early cognitive disadvantages (Song & Luo, 2024). Empirical evidence shows that positive teacher–student interactions can help children with early cognitive disadvantages gradually strengthen their reasoning, problem-solving, and reflective thinking in their everyday learning process (Elsaesser et al., 2018; Sankalaite et al., 2023; Shi et al., 2025). By offering consistent academic guidance together with emotional support, teachers help students improve their cognitive development, which in turn may support more upward mobility across generations (Riordan, 2022; Zheng et al., 2023).

China has long faced marked disparities in the distribution of educational resources between urban and rural areas (Zheng et al., 2023). In many rural areas, insufficient public funding and a persistent lack of well-trained teachers have increased the disparity in the cognitive development of rural children in comparison to their urban peers (Y. Lu et al., 2024). At the same time, many rural families continue to experience economic hardship that limits the educational opportunities they can offer their children (Li et al., 2025). Taken together, these interrelated disadvantages have perpetuated what scholars describe as the intergenerational reproduction of cognitive poverty (Schulz et al., 2017).

To address these longstanding disparities, the Chinese government launched a range of institutional initiatives to strengthen the rural teaching workforce, among which the Rural Teacher Support Program (RTSP) was the most extensive. Implemented from 2015 to 2020, the program aimed to enhance both the quantity and quality of teachers in under-resourced rural schools through expanded recruitment, increased living subsidies, and improved in-service training, thereby building a high-quality teaching workforce committed to long-term rural service. The program designated the “lao-shao-bian-qiong” areas in Central and Western China as priority regions, an official term referring to resource-constrained and relatively impoverished areas, and primarily targeted teachers in rural compulsory education, namely primary and lower-secondary schools. Its core measures sought to expand the rural teacher supply through multiple channels, including broadening recruitment pathways, strengthening locally oriented preparation, and mobilizing retired teachers for short-term rural service. In parallel, it aimed to attract high-performing urban teachers to rural schools by improving pay and welfare protections for rural teachers, and to enhance teachers’ professional capacity through diverse forms of training and professional development. The program was implemented across subjects in rural compulsory education schools rather than being discipline-specific, while also prioritizing hard-to-staff posts and subject areas with persistent shortages.

China’s central and local governments conducted annual special supervisory inspections of the RTSP’s implementation. According to the official statistics of the Ministry of Education (MOE), by the end of 2020, the RTSP had been fully implemented in 725 counties formerly classified as contiguous poverty-stricken counties across 22 central and western provinces. The program provided living subsidies to teachers in roughly 80,000 rural schools and reached more than 1.29 million rural teachers. Publicly available official materials primarily document program implementation and coverage, rather than providing a national impact evaluation that directly assesses effects on students’ cognitive outcomes. In this context, the program’s gradual implementation across counties created regional variation in teacher-related conditions, offering a quasi-natural context for examining how such changes relate to students’ cognitive development (Song & Luo, 2024).

While the implementation of such institutional initiatives offers a valuable opportunity to explore how changes in teacher-related conditions are linked to students’ cognitive development, their cognitive implications remain underexplored. Prior research has largely conceptualized teacher support at a micro level, focusing on teacher training and professional development. By comparison, large-scale, supply-side institutional support, such as the RTSP, has received less attention, particularly teacher quantity and staffing adequacy. At the same time, student outcomes in the literature are more often measured by academic achievement, with fewer studies directly examining broader cognitive development outcomes. Evidence on whether teacher training support improves students’ academic performance has also been mixed. Some studies suggest that in-service teacher training can improve instructional skills and strengthen teachers’ professional confidence, which may translate into better learning outcomes for students (Desimone et al., 2002; Ross & Bruce, 2007). Similarly, the academic performance of rural students has been reported to be increased through training programs, especially those in the intermediate or higher level (Sun & Du, 2021). Targeted technical training for rural teachers can further enhance student participation and achievement in settings with limited resources (G. Zhang, 2025). However, other studies offer a less optimistic view. They state that the teacher training does not usually result in any significant changes in the knowledge or attitudinal changes and classroom practices of the teachers. In some cases, it has little direct effect on students’ academic outcomes (Estudillo et al., 2023; Loyalka et al., 2019). Consistent with this mixed evidence, an evaluation of China’s National Teacher Training Program (NTTP) produced similar findings; although the program improved teachers’ mathematical knowledge, it did not significantly alter their teaching practices or students’ mathematics scores (M. Lu et al., 2019). As a result, it is still unclear whether large-scale, supply-side institutional support for rural teachers is associated with students’ cognitive development, a broader and more foundational dimension of human capital formation. Whether such support operates not only through staffing adequacy but also through students’ perceived classroom experience, as reflected in their satisfaction with teachers, is also understudied.

Building on these insights, the present study explores how institutional improvements in teacher-related conditions relate to students’ cognitive development. It further investigates two mechanisms that may account for this link: teacher quantity and students’ satisfaction with teachers.

### 1.1. Teacher Quantity and Students’ Cognitive Abilities

The number of teachers is one of the main mechanisms that link institutional support for rural teachers to students’ cognitive development. Having an adequate number of competent teachers allows for more space for individualized instruction and richer cognitive engagement. These conditions have been repeatedly shown to nurture cognitive growth. (Rakesh et al., 2024; Shemshack & Spector, 2020). However, the fact that there has always been a lack of qualified teachers in rural China has limited cognitive stimulation and quality face-to-face classroom interaction (Jiang & Yip, 2024). When staffing levels improve, schools may be better positioned to lower the student–teacher ratio and increase students’ instructional exposure, including more time on tasks and greater potential for individualized attention and timely feedback. Such interaction helps them sustain attention, improve working memory, and refine problem-solving skills, which are key components of cognitive functioning (S. Guo et al., 2023).

A growing body of research lends support to this association. Reductions in student–teacher ratios and greater teacher stability have been linked to improvements in both academic learning and cognitive performance (Bennell, 2022; Nakamura & Dev, 2022). From a human capital perspective, teachers act not only as transmitters of knowledge but also as social role models who nurture reasoning and metacognitive awareness. Through this process, they help students develop sustained cognitive growth (Jokela, 2022). In rural contexts where cognitive support from families is scarce, higher teacher density may compensate for limited home-based learning inputs by providing sustained cognitive stimulation (Y. Zhou et al., 2023). Accordingly, increases in teacher quantity are expected to be positively associated with students’ cognitive development, which can translate into greater instructional access and teacher support for students.

Between 2015 and 2020, the RTSP included measures that plausibly influenced rural staffing levels. Beyond expanding recruitment channels, the program emphasized mechanisms to increase and stabilize teacher supply in rural compulsory education schools, including strengthening the Rural Teacher Special Post Program, encouraging targeted local preparation, facilitating teacher mobility and rotation within counties, and aligning staffing quotas for rural schools with urban standards. These measures were intended to address persistent shortages in hard-to-staff rural schools and to improve staffing adequacy at the county level (MOE, 2015). Given these design features and data availability, we treat teacher quantity as a measurable proxy for staffing adequacy at the county level and as a key indicator that is likely to expand students’ opportunities to learn. In our empirical analyses, we examine whether RTSP exposure is associated with changes in teacher staffing, while the relationship between teacher quantity and cognitive development is discussed theoretically based on prior research.

### 1.2. Students’ Satisfaction with Teachers and Their Cognitive Abilities

Another important mechanism connecting institutional support for rural teachers to students’ cognitive development is students’ satisfaction with their teachers. In this study, students’ satisfaction with teachers is intended to capture students’ perceived teacher support and the quality of day-to-day teacher–student interactions. Teachers in under-resourced rural schools often face poor working conditions with limited opportunities for professional advancement and relatively low wages, which may affect both their motivation and quality of instruction (Zhao, 2024; Long et al., 2025).

Developmental and educational psychology studies indicate that teachers who provide consistent emotional support and responsive instruction can better meet students’ learning needs. Such practices can be used to make classroom experiences more meaningful, particularly in environments with few resources (J. Wang et al., 2021; Zheng et al., 2023). When students perceive greater teacher effort in academic support, emotional care, and classroom feedback, they generally report a greater sense of satisfaction with their teachers (J. Wang et al., 2022). A stronger sense of satisfaction can enhance learning motivation and behavioral engagement, forming an important pathway through which supportive teacher–student relationships contribute to cognitive development (S. Zhou et al., 2023; W. Zhang & Hu, 2025; L. Zhou et al., 2022).

Between 2015 and 2020, the RTSP included measures that plausibly influenced rural teachers’ working conditions and professional support. The program combined differentiated living subsidies and welfare protections with measures to strengthen professional development, including large-scale, in-service training and more supportive promotion pathways that were explicitly tilted toward rural posts (MOE, 2015). These measures aimed to raise teacher morale and instructional engagement. In doing so, they might enhance students’ perceptions of teacher support. Accordingly, students’ satisfaction with teachers is treated in this study as a plausible and mechanism-consistent channel through which the RTSP may relate to enhanced cognitive ability, and we avoid interpreting it as a separately identified causal mediation in the current data.

Beyond these mechanisms, the effects of teacher-related supports on students’ cognitive development may vary across different groups.

### 1.3. Gender Differences

In many parts of rural China, traditional son-preference norms in many rural areas have led families to invest more in boys (Chu et al., 2007). As a result, girls often have fewer learning opportunities and slower cognitive progress. (Demirel-Derebasoglu & Okten, 2022; Yang & Wu, 2023). Because of these persistent disparities, the benefits of educational improvement programs may not be distributed evenly across genders. Compared with boys, girls generally receive less academic support from their families and thus depend more on school resources for cognitive development (Tong & Li, 2024).

From the standpoint of developmental psychology, girls’ greater sensitivity to instructional and emotional cues may make them more responsive to teacher support (Kim et al., 2024; J. Liu, 2021). This heightened sensitivity may make girls more responsive to improved teacher support. In settings where family educational support is weak, such institutional reforms could lead to stronger cognitive gains for girls (W. Guo & Zhou, 2021; W. Guo, 2024). In other words, girls’ reliance on external educational resources may strengthen the connection between enriched teaching environments and cognitive growth.

Despite these theoretical insights, little empirical work has tested whether the cognitive outcomes of teacher-related improvements vary by gender. The present study therefore examines whether rural teacher support is differently associated with boys’ and girls’ cognitive development.

### 1.4. Household Income Differences

Beyond gender, socioeconomic background may also shape how rural educational improvements influence students’ cognitive development. A substantial amount of empirical work indicates that children’s cognitive development is closely related to household economic conditions (Sosu & Schmidt, 2022). Compared with their wealthier peers, students from low-income households tend to perform worse in verbal comprehension, mathematics, and logical reasoning (Cooper & Stewart, 2021; Dickerson & Popli, 2016). Because their families face financial constraints and limited access to extracurricular learning opportunities, these students often experience disadvantages in early academic achievement and cognitive growth (Li et al., 2023). In rural regions with scarce resources, they depend heavily on school-based support to make up for insufficient home investment in education (L. Zhou et al., 2020).

Drawing on the theory of diminishing marginal returns, public educational investment is expected to generate larger benefits for groups starting from lower baselines (X. Xie et al., 2023). As a result, improvements in teacher-related resources under programs such as the RTSP could yield greater cognitive gains for students from low-income households. Even so, previous studies warn that elite capture in targeted education initiatives may concentrate resources among advantaged groups and limit the benefits available to disadvantaged students (Bauchet et al., 2015; Song et al., 2022; Cheng et al., 2022).

Given these mixed possibilities, this study assesses whether the link between rural teacher support and students’ cognitive abilities differs across different levels of household income.

### 1.5. The Present Study

Long-term limitations in rural educational resources have contributed to widening cognitive differences between rural and urban students. To mitigate this challenge, institutional initiatives such as the RTSP were launched to strengthen the rural teaching workforce through recruitment expansion, financial incentives, and professional development. Yet the empirical evidence has largely concentrated on training-based support and academic achievement. It therefore remains unclear whether institutional support for teachers, including staffing improvements, is associated with students’ broader cognitive development, and whether such links reflect both staffing conditions and students’ classroom experience.

This study aims to address this gap by examining whether rural teacher support is associated with students’ cognitive ability in rural China. Using longitudinal data from the China Family Panel Studies (2012–2020), we employ a difference-in-differences (DID) approach with individual and county-fixed effects to estimate the association between exposure to the RTSP and students’ cognitive outcomes.

We further examine two pathways through which the RTSP may be associated with students’ cognitive development. The first is a structural pathway that aligns with the RTSP’s staffing oriented measures and is captured by teacher quantity and staffing adequacy. The second is a relational and experiential pathway that aligns with the RTSP’s incentive and professional support measures and is captured by students’ satisfaction with teachers, which reflects students’ perceived classroom experience and support.

Based on the above reasoning, we advance the following hypotheses:

**Hypothesis 1 (H1).**  *Exposure to the RTSP is positively associated with improvements in students’ cognitive ability*.

**Hypothesis 2 (H2).**  *The RTSP enhances students’ cognitive outcomes by increasing the number of rural teachers*.

**Hypothesis 3 (H3).**  *The RTSP promotes students’ cognitive development by improving students’ satisfaction with teachers*.

**Hypothesis 4 (H4).**  *The positive relationship between the RTSP and students’ cognitive outcomes is more pronounced among female students*.

**Hypothesis 5 (H5).**  *The positive relationship between the RTSP and students’ cognitive outcomes is more pronounced among students from low-income households*.

## 2. Methods

### 2.1. Sample and Procedure

This study draws on data from the China Family Panel Studies (CFPS), a nationally representative longitudinal survey of Chinese households run by the Institute of Social Science Survey at Peking University (Y. Xie & Hu, 2014). The CFPS collects rich information on individuals’ demographic characteristics, educational experiences, and psychological and cognitive development through biennial face-to-face survey interviews conducted by trained CFPS interviewers. Information in full detail about the survey design and data access can be accessed at http://www.isss.pku.edu.cn/cfps/en/ (accessed on 13 January 2026). How the data was used, including the process of determining the sample, any exclusions, and all measures applied in the study, is reported below.

The analytic sample includes rural children aged 10–16, a developmental stage during which cognitive ability remains highly malleable and responsive to environmental conditions. To maintain temporal consistency and allow for within-individual comparisons, the analysis retained only those participants with at least four valid records across five CFPS survey waves (2012–2020). Each participant was required to have at least one observation before and one after the introduction of the RTSP in 2015. Records missing county identifiers were omitted. After data cleaning, the final sample comprised 2996 child-year observations.

Because the RTSP targeted less-developed regions of China officially defined as “lao-shao-bian-qiong” areas, we adopted the regional classification framework proposed by Fang and Zhao (2021). Counties identified as “Contiguous Poverty-Stricken Areas” in the National Rural Poverty Alleviation and Development Program Outline (2011–2020) were assigned to the treatment group, while counties not designated under this category served as the comparison group. Among the 2996 retained observations, 1056 were from treatment counties, and 1940 were from control counties.

The DID approach is particularly well-suited for evaluating intervention effects when randomized experiments are not feasible (Imbens & Wooldridge, 2009). By leveraging quasi-experimental designs, DID helps estimate differential changes associated with an intervention by comparing outcome trends between treated and comparison groups (Card & Krueger, 1994; Bertrand et al., 2004). This method accounts for variations between treatment and control groups, as well as conditions before and after policy implementation (Angrist & Pischke, 2014), leading to the construction of a counterfactual framework that contributes to the study.

The RTSP was launched nationwide in 2015 and prioritized counties in nationally designated Contiguous Poverty-Stricken Areas, providing a quasi-natural experimental setting to examine the relationship between rural teacher support and students’ cognitive outcomes. A simple before–after comparison may conflate the program’s contribution with contemporaneous macro shocks, differential regional trends, and other policy changes, thereby yielding biased estimates. Therefore, we treat the RTSP as a quasi-natural experiment and apply the DID approach to compare changes in cognitive performance between treatment and comparison groups. Because the CFPS is conducted biennially, we define the pre-intervention period as 2012 and 2014 and the post-intervention period as 2016, 2018, and 2020. Identification relies on the assumption of comparable pre-intervention trends between the two groups, which we assess through a parallel-trend test and robustness test in the subsequent analyses. In addition, to further improve comparability between treatment and comparison counties on observable pre-intervention characteristics, we implement propensity score matching difference-in-differences (PSM-DID) as a robustness check and re-estimate the DID on the matched sample.

In addition, we include several county-level macroeconomic variables, including per capita GDP, the share of secondary and tertiary industries, and per capita fixed-asset investment. These variables were obtained from municipal and county statistical yearbooks covering 2012–2020. The final dataset covers 142 counties, comprising 29 treatment counties and 113 comparison counties.

### 2.2. Variables

#### 2.2.1. Dependent Variables

Students’ cognitive ability serves as the dependent variable in this study. The measure comes from the cognitive testing module of the CFPS, which administered two alternating test batteries across waves: Set A focused on character recognition and basic arithmetic, whereas Set B assessed word recall and number series reasoning. To make scores comparable across waves, results from the four subtests were combined into two broader indicators: a vocabulary score (recognition and recall) and a math calculation test score (calculation and series).

The vocabulary and math tests primarily capture crystallized cognitive skills related to accumulated knowledge and formal schooling, whereas the recall and number series tasks reflect fluid cognitive processes, including working memory and abstract reasoning. Following J. Wu et al. (2022), the combined score of the vocabulary and math composites is adopted as a unified indicator of students’ cognitive ability. To remove interwave differences in test administration and difficulty, the total score is z-standardized within each survey year (M = 0, SD = 1). This procedure yields a standardized cognitive ability index that is comparable across survey waves and interpretable in standard deviation units.

#### 2.2.2. Mechanism Variables

This study selects the number of teachers and students’ satisfaction with teachers as mechanism variables. Teacher quantity represents the structural dimension of educational support, reflecting the extent of teacher resources available to students in each county. Following Song et al. (2022), teacher quantity is measured as the ratio of the total number of primary and secondary school teachers in a region to the total registered population at the end of the year. Students’ satisfaction with teachers captures the perceptual dimension of teacher support. This variable measures students’ subjective evaluation of their teachers’ ethics, competence, and classroom interactions. Following Z. Liu (2023), this study adopts the measure from the CFPS questionnaire, specifically from students’ responses to the item on their satisfaction with their homeroom teacher. In the Chinese compulsory schooling context, homeroom teachers typically also teach at least one subject and play an important role in organizing classroom routines and learning support, which makes this measure a reasonable proxy for students’ perceived teacher support in daily schooling. Participants rated their satisfaction on a five-point Likert scale, where a higher score reflected a stronger sense of satisfaction. For analysis, responses scoring 3 or above were treated as “satisfied” (coded as 1), and those below 3 were treated as “not satisfied” (coded as 0).

#### 2.2.3. Independent Variables

The independent variable in this study is exposure to the Rural Teacher Support Program (RTSP). This nationwide policy initiative, introduced in 2015, was designed to strengthen the rural teaching workforce by offering financial incentives, in-service professional training, and better working conditions. Through these measures, the program aimed to enhance both the quantity and quality of rural teachers while fostering more supportive classroom environments for students in under-resourced areas.

To assess how teacher support is associated with students’ cognitive outcomes, the study employs a quasi-experimental difference-in-differences (DID) framework. It compares changes in cognitive performance over time between counties covered by the RTSP and those outside the program. Specifically, the treatment group includes counties designated as key RTSP implementation areas, whereas the comparison group includes regions outside the RTSP coverage. The RTSP effect is identified using the interaction of two dummy variables: ${treat}_{c}$, which equals 1 if a county belongs to the treatment group and 0 otherwise; and ${treat}_{t}$, which equals 1 for observations after 2015 and 0 otherwise. The interaction term ${treat}_{c}$ × ${treat}_{t}$ represents the quasi-experimental estimate of the RTSP’s effect on children’s cognitive ability.

#### 2.2.4. Control Variables

To ensure the robustness of the findings and reduce the influence of omitted variables, this study incorporates a comprehensive set of controls at the county, individual, and family levels, following prior research on education policy evaluation (Schlotter et al., 2011).

County-level controls account for regional economic development and industrial structure, both of which may influence the allocation of educational resources and parental investment in children’s learning. These variables include per capita GDP, per capita fixed-asset investment, and the shares of secondary and tertiary industries. Prior research has shown that regional GDP is closely related to educational resource availability (Hanushek & Woessmann, 2012) and that spatial disparities in education and health are linked to economic and structural inequalities (X. Zhang & Kanbur, 2005).

Individual-level controls capture demographic and personal attributes that may influence cognitive outcomes, including gender, age, health status, and years of education. Gender is included as a control variable because social and cultural expectations often influence the kinds of learning opportunities available to students (Demirel-Derebasoglu & Okten, 2022). Age is also controlled, as it relates to the ongoing development of cognitive skills through neurological maturation and accumulated learning experiences (M.-T. Wang et al., 2021). Health status reflects physical and psychological conditions affecting learning capacity (Shaw et al., 2015). Years of education represent prior exposure to educational environments that contribute to cognitive development (Y. S. Zhang et al., 2024).

Family-level controls address socioeconomic heterogeneity in children’s learning environments. In line with the established role of family capital in shaping educational outcomes (Hou et al., 2023), we include total household income and housing assets. Income reflects the family’s ability to provide learning resources and support, while housing assets serve as a proxy for long-term wealth and socioeconomic stability, both of which are associated with enriched home learning environments and access to quality education (Li et al., 2023; Wiborg & Grätz, 2022).

The description of each variable is presented in Table 1.

### 2.3. Empirical Model

To estimate the causal impact of the RTSP on students’ cognitive ability, this study adopts a difference-in-differences (DID) approach, which compares within-individual changes in outcomes between treatment and comparison groups before and after the policy intervention. This quasi-experimental design helps isolate the policy’s net effect by controlling for both time-invariant regional heterogeneity and common temporal shocks (Angrist & Pischke, 2014). Following prior studies on education and poverty alleviation (Song et al., 2022), the year 2015 is defined as the intervention point, given the policy’s nationwide launch, and the analytic sample spans 2012–2020. The baseline model is specified as follows:

(1) $$Y_{ict}=\alpha +\beta {treat}_{c}*{treat}_{t}+\zeta {control}_{ict}+\theta_{t}+\gamma_{c}{\;+\;\varepsilon }_{ict}$$

where *i*, *c*, and *t* denote the individual, county, and year, respectively. $Y_{ict}$ denotes the dependent variable, which is students’ cognitive ability.$\;{treat}_{c}$ is a county-level binary indicator equal to 1 if county *c* belongs to the designated treatment group (concentrated poverty-stricken counties), and 0 otherwise. ${treat}_{t}$ is a time dummy that equals 1 for the post-intervention period (2015–2020) and 0 for the pre-intervention period (2012–2014). ${control}_{ict}$ denotes a set of control variables at the individual, family, and county levels, including gender, age, health status, years of education, per capita GDP, per capita fixed-asset investment, the shares of secondary and tertiary industries in GDP, household total income, and housing assets. $\theta_{t}$ and $\gamma_{c}$ denote year- and county-fixed effects, respectively. Year-fixed effects absorb common shocks over time that affect all counties simultaneously (e.g., macroeconomic fluctuations), while county-fixed effects control for time-invariant heterogeneity across counties such as geography and historical legacy. By combining county-fixed effects with time-varying county-level controls, the model accounts for both unobserved heterogeneity that is constant over time and observable structural characteristics that evolve over time. The idiosyncratic error term is denoted by $\varepsilon_{ict}$.

The key coefficient of interest, β, represents the estimated impact of the RTSP on students’ cognitive ability. A positive and statistically significant β indicates that exposure to the program is associated with improvements in children’s cognitive performance, suggesting that enhanced teacher support contributes to cognitive development in under-resourced rural areas.

## 3. Results

### 3.1. Baseline Estimated Results

The baseline DID estimates are reported in Table 2. Column (1) controls for county-fixed effects, year-fixed effects, and county- and individual-level covariates. Column (2) additionally incorporates family-level covariates, including total household income and housing assets, to account for household socioeconomic background and assess robustness, given that prior research suggests family socioeconomic conditions are associated with children’s cognitive development and educational outcomes (Hou et al., 2023; Li et al., 2023; Wiborg & Grätz, 2022). In both specifications, exposure to the RTSP is positively and significantly associated with students’ cognitive ability at the 1% level. The estimated effect increases slightly from 0.168 to 0.185 standard deviations after controlling for household socioeconomic conditions, indicating that the effect is robust to the inclusion of family-level factors. The magnitude of the coefficient suggests that the RTSP contributes to a meaningful improvement in children’s cognitive performance in under-resourced rural areas. Overall, these findings provide strong empirical support for Hypothesis 1, which posits a positive association between the RTSP and students’ cognitive ability.

### 3.2. Parallel Trend Test

A core identification requirement of the DID approach is the parallel trends assumption, which posits that treatment and comparison groups would have followed similar outcome trajectories in the absence of the intervention (Becker, 1983; Jacobson et al., 1993). To empirically assess this assumption, we estimate an event-study specification of the following form:

(2) $$Y_{ict}=\alpha +\sum_{t\neq 2014}\;\beta_{t}{treat}_{c}\;D_{t}+\zeta {control}_{ict}+\theta_{t}+\gamma_{c}{\;+\;\varepsilon }_{ict}$$

where $D_{t}$ represents year dummies, and $\beta_{t}$ captures the dynamic differences in students’ cognitive ability between treatment and control counties relative to the baseline year 2014, after controlling for county- and individual-level covariates and fixed effects. If the coefficients $\beta_{t}$ are statistically insignificant for *t* ≤ 0 (from 2012 to 2014), the parallel trends assumption is satisfied.

**Figure 1:**
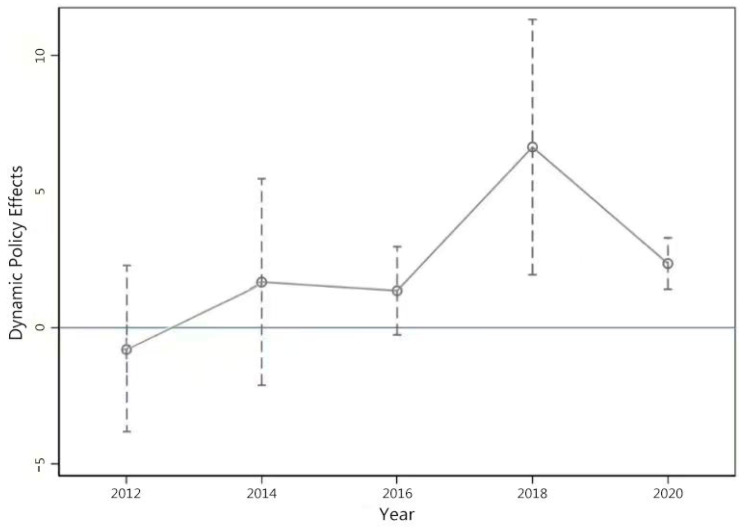
Dynamic policy effects and test of the parallel trends assumption.

Figure 1 presents the estimated dynamic effects. Prior to the implementation of the policy, the coefficients were close to zero, and the confidence intervals included zero, suggesting that the treatment and control groups followed similar trends before the program, which is consistent with the parallel trends assumption. After 2014, the estimated coefficients from 2015 to 2018 show a gradual increase, while the coefficient in 2020 declines but remains above zero. This pattern is consistent with the interpretation that the RTSP contributed to improvements in students’ cognitive ability.

### 3.3. Robustness Test

#### 3.3.1. Propensity Score Matching Difference-in-Differences

To enhance the comparability between treatment and comparison groups and reduce potential sample selection bias, we apply a propensity score matching (PSM) approach prior to the DID estimation. Specifically, following Austin (2011), Morgan et al. (2009), and Van Helvoort-Postulart et al. (2009), the propensity scores are estimated using a logit model based on both county-level and individual-level covariates observed in 2014, the year before the RTSP implementation.

The county-level variables include per capita GDP, the shares of secondary and tertiary industries, and per capita fixed-asset investment, while the individual-level variables include gender, age, health status, and years of education. One-to-two nearest-neighbor matching without replacement is employed, with a caliper width of 0.01, to ensure close matches between treatment and comparison units. Table 3 reports the standardized mean differences in covariates before and after matching. All covariates exhibit standardized mean differences below 10% after matching, indicating that the balance condition is satisfied and that the matched sample achieves adequate comparability.

Using this matched sample, we re-estimate the policy effect through a DID model (Table 4). The estimated coefficient for the RTSP remains highly significant at the 1% level and close in magnitude to the baseline estimate, reinforcing the robustness of the program’s positive effect on students’ cognitive ability.

#### 3.3.2. Accounting for Overlapping Policy Effects

To further ensure robustness, we control for potential confounding effects of other overlapping policies during the study period (Zhong et al., 2021). In 2012, the Chinese government revised the list of national key poverty-stricken counties, a targeted poverty alleviation initiative that provided additional fiscal transfers and development support to designated underdeveloped areas. This revision included several counties classified as Contiguous Poverty-Stricken Areas in the National Rural Poverty Alleviation and Development Program Outline (2011–2020), some of which later became part of the RTSP coverage. To isolate the independent impact of the RTSP and account for potential policy overlap, we include interaction terms between a binary indicator for national key poverty-stricken counties and year dummies in the DID model. The re-estimated results, presented in Table 5, yield a coefficient of 0.180, which remains significant at the 1% level. This finding indicates that the RTSP’s positive association with students’ cognitive abilities is robust in both magnitude and significance, reinforcing the validity and stability of the baseline results.

### 3.4. Mechanism Analysis

#### 3.4.1. Teacher Quantity

As proposed in Hypothesis 2, this section examines teacher quantity as a key mechanism through which the RTSP influences students’ cognitive development. Teacher quantity is measured as the ratio of the total number of primary and secondary school teachers to the county’s year-end population, thereby controlling for differences in population size and ensuring comparability across counties and over time. This relative indicator more accurately reflects the adequacy of teacher resources available to the local population. Using this indicator as the dependent variable, we estimate a county-level panel DID model. The results, reported in Table 6, indicate a statistically significant positive coefficient at the 1% level, suggesting that the RTSP led to a marked increase in teacher quantity in treatment counties relative to comparison counties. This finding supports the hypothesis that the program strengthened local educational human resources, thereby indirectly facilitating students’ cognitive improvement, confirming Hypothesis 2.

#### 3.4.2. Students’ Satisfaction with Teachers

As proposed in Hypothesis 3, this section examines whether the RTSP enhanced students’ satisfaction with their teachers, which is conceptually linked to their cognitive engagement and learning motivation. Using an individual-level panel DID model, the results reported in Table 7 show a coefficient that is significantly positive at the 10% level. Specifically, following the implementation of the program, students’ satisfaction with teachers increased by approximately 1.6%. This suggests that the RTSP improved students’ satisfaction with their teachers, thereby indirectly enhancing students’ cognitive development and confirming Hypothesis 3.

### 3.5. Heterogeneity Analysis

#### 3.5.1. Heterogeneity in Gender

Prior studies suggest that persistent gender disparities in rural education, shaped by traditional norms and constrained household resources, may limit girls’ educational opportunities and hinder their cognitive development (Demirel-Derebasoglu & Okten, 2022; Yang & Wu, 2023). Compared with boys, girls may receive less educational support at home and thus tend to depend more on school-based resources for cognitive growth (Tong & Li, 2024). Given these disparities, the RTSP could be expected to generate stronger cognitive benefits for female students.

To test this hypothesis, the sample is divided by gender, and separate regressions are conducted for male and female students. The results (Table 8) indicate that the RTSP increases the cognitive ability of female students by 0.251 standard deviations, and the effect is statistically significant at the 5% level. In contrast, the estimated improvement for male students is 0.094 standard deviations and does not reach statistical significance.

Overall, these findings suggest that the positive effect of the RTSP on students’ cognitive ability is largely driven by improvements among female students. This pattern supports Hypothesis 4. This result is in line with the literature emphasizing girls’ relative disadvantage in rural education and aligns with the theoretical expectation that educational interventions yield greater benefits for groups with initially limited access to resources.

#### 3.5.2. Heterogeneity in Household Total Income

Prior research indicates that household income substantially influences students’ educational outcomes, with children from low-income families facing higher risks of educational inequality and limited academic opportunities (Li et al., 2023). To test whether the RTSP benefits less advantaged students, we divide the 2996 observations into low- and high-income households using the sample mean of total household income as the threshold. The low-income group includes 2079 observations, and the high-income group includes 917 observations.

Results from separate regressions (Table 9) show that the RTSP effect on students’ cognitive ability is weak and statistically insignificant among high-income households but strong and positive at the 1% level among low-income households. These findings indicate that students from low-income families are more likely to benefit from the RTSP, supporting Hypothesis 5. This result aligns with the theoretical expectation that educational interventions yield higher marginal returns for disadvantaged groups while mitigating concerns that program resources might have been disproportionately captured by higher-income households.

## 4. Discussion

This study offers longitudinal evidence on how teacher-related institutional support, exemplified by China’s Rural Teacher Support Program, relates to students’ cognitive development in rural settings. Based on nationally representative panel data, the results consistently show that teacher support is linked to improvements in children’s cognitive development in resource-constrained settings. The association is still retained even when robustness checks and alternative specifications have been implemented. The mechanism analyses suggest that improvements in teacher quantity and students’ satisfaction with teachers are two plausible pathways through which the RTSP may be related to cognitive development. In addition, the heterogeneity analysis reveals that such associations differ significantly in gender and household income groups. While the patterns are robust across specifications, unobserved time-varying confounding cannot be fully excluded. Accordingly, the estimates should be interpreted as associations derived from a quasi-experimental setting rather than as definitive causal effects. These findings are broadly consistent with human capital theory (Becker, 1994) and educational inequality models (Coleman, 1988) and reflect the importance of institutional interventions in elucidating structural disadvantages and enhancing cognitive development.

The findings highlight two pathways through which teacher support may relate to students’ cognitive development. These mechanisms are theoretically salient because staffing conditions shape instructional interaction opportunities, students’ perceived teacher support reflects relational and motivational dimensions relevant to cognitive development (Krueger, 1999; Roorda et al., 2011), and they map closely onto the RTSP’s core instruments. First, the program is associated with increases in teacher numbers in rural schools, hence lowering student-to-teacher ratios and increasing their chances at individualized instruction, which improves cognitive stimulation and self-regulated learning (Bennell, 2022; Nakamura & Dev, 2022; Y. Zhou et al., 2023). Second, the RTSP is also linked to higher student satisfaction with teachers in terms of both instructional quality and socio-emotional support (J. Wang et al., 2022). When students feel more care and feedback, they demonstrate greater engagement in learning and academic persistence, which are commonly linked to cognitive development (S. Zhou et al., 2023).

Importantly, other mechanisms such as instructional quality, classroom practices, or pedagogical innovation may also be associated with children’s cognitive development. However, these classroom-level processes are not observed in a consistent, longitudinal manner in the available data, which prevents a direct test of these channels in the current study. Accordingly, we focus on teacher quantity and students’ satisfaction with teachers because they can be measured most consistently and comparably over time. As a result, the mechanism evidence should be interpreted as partial rather than exhaustive, and the omission of classroom-level measures means that the two tested pathways cannot be read as exclusive mechanisms. Instead, they may partly reflect correlated, unmeasured classroom processes that evolve alongside staffing conditions and students’ perceived teacher support.

Besides the core mechanisms, gender differences offer additional insight into how institutional and cultural contexts influence cognitive development. The association between teacher-related institutional support and cognitive outcomes appears stronger among girls than among boys. This trend aligns with past studies that have revealed that gender inequality in rural China has been persistent, with gender-based norms and rural household practices traditionally privileging the education of boys (Yang & Wu, 2023).

Thus, girls are more likely to rely on the resources offered in schools to fill gaps left by ineffective family support (Huang et al., 2023). Girls can be especially well positioned to have an advantage when there is attention to the supply of teachers, as well as the improvement of teacher–student relationships. In the wider cultural background, Confucian traditions have traditionally paid more attention to the education of boys, which has placed girls in a rather disadvantaged situation (Yan & Ren, 2019). On the whole, these results imply that teacher–institutional changes can be used to play a compensatory developmental role in reducing gender disparities in cognitive development in conditions of resource limitations. In policy terms, this pattern highlights that strengthening teacher supply and teacher–student support in rural schools may help buffer gender-linked disadvantages in learning opportunities. Related evidence from rural India suggests that greater access to female teachers is associated with narrower gender gaps in learning outcomes, highlighting the potential relevance of gender-responsive teacher deployment in disadvantaged areas (Muralidharan & Sheth, 2016).

The associations of the teacher support program among low-income-household students also seem to be stronger than among high-income-household students. Consistent with prior research, children from low-income families often receive less cognitive stimulation and enrichment at home and consequently draw more of their motivational and learning support from teachers and the school environment (Y. Wu & Zhang, 2024). Conversely, children in wealthier households enjoy more opportunities of individual tutoring, the Internet, and accommodating learning conditions that cushion them against disparities at the school level. Such observations are in line with the concept of diminishing marginal returns to human capital investment implying that teacher-related institutional improvements yield greater developmental returns among students starting from lower baselines (Dai & Li, 2022; Heckman, 2006). In that regard, teacher-centered institutional support may function as a leveling developmental factor supporting cognitive development for children facing tighter socioeconomic constraints. This pattern resonates with broader discussions of socioeconomic gradients in human capital formation, in which school-based inputs may be relatively more salient when home learning resources are limited. In terms of policy design, evidence syntheses on staffing disadvantaged and hard-to-staff schools emphasize the importance of equity-oriented targeting and bundled deployment supports to improve teacher availability and stability in high-need schools (Evans & Acosta, 2023; See et al., 2020). Related evidence also suggests that improved staffing conditions can be particularly salient for disadvantaged students, although effectiveness depends on implementation quality and local constraints (Schanzenbach, 2014).

The implications of these findings speak to human capital theory (Becker, 1994; J. Liu, 2021) by suggesting that teacher-centered institutional support is associated with students’ cognitive development through both structural improvements in staffing and socio-emotional dimensions of support in resource-constrained settings. They also contribute to educational inequality research (Coleman, 1988; Xu, 2023) by suggesting that improvements in staffing levels and perceived teacher support may be associated with reduced disadvantages in cognitive development linked to gender norms and socioeconomic constraints. From a policy perspective, the findings point to the importance of differentiated teacher support policies that consider local developmental requirements rather than uniform prescriptions. In particular, heterogeneity by household income and gender suggests that equity-oriented targeting may be important, prioritizing hard-to-staff schools and focusing on groups facing weaker home learning inputs. Strengthening supportive teacher–student relationships and socio-emotional classroom climates, and, where relevant, adopting gender-responsive deployment approaches, including improving access to female teachers in disadvantaged areas, may further advance equity-oriented goals (Evans & Acosta, 2023; See et al., 2020; Muralidharan & Sheth, 2016). Taken together, these implications also highlight the value of large-scale, bundled teacher support, as exemplified by the RTSP. Targeted packages that jointly improve staffing adequacy and supportive classroom experiences may be especially important for strengthening cognitive development among disadvantaged students and advancing educational equity. Overall, these implications may be informative for other developing country settings with comparable rural teacher shortages and persistent inequalities in learning opportunities, while recognizing that local institutional conditions may shape implementation and effectiveness.

## 5. Limitations

Despite the strengths, several limitations warrant consideration. First, our mechanism analyses examine teacher quantity and students’ satisfaction with teachers because these constructs are consistently available and comparable over time in the current data. Other plausible channels, including instructional quality and classroom practices, cannot be tested directly due to the lack of consistent longitudinal measures. Therefore, the mechanism evidence should be interpreted as partial rather than exhaustive, and this data constraint limits how specifically the estimated association can be attributed to any single mechanism. The two tested channels may also capture changes that co-occur with unmeasured classroom processes.

Second, although the difference-in-differences design and robustness checks mitigate some confounding concerns, the analysis remains observational, and unobserved time-varying factors cannot be fully ruled out. Accordingly, the estimates are best interpreted as quasi-experimental associations rather than definitive causal effects.

Third, measurement choices may affect validity and comparability. Cognitive ability was constructed from an alternating CFPS test module and standardized within each survey year, which may be sensitive to the aggregation and standardization strategy. In addition, students’ satisfaction with teachers is dichotomized in the main analyses, which may reduce information and introduce cutoff sensitivity. Moreover, this measure reflects satisfaction with the homeroom teacher rather than subject-specific instructional quality, so mechanism-related interpretations should be made cautiously.

Finally, this study was conducted in the context of rural China and is anchored in the design and targeting rules of the RTSP. Because the CFPS sample and the institutional features of the RTSP are China-specific, the extent to which the estimated associations generalize to other contexts may depend on local teacher labor markets, fiscal capacity, and implementation conditions. Differences in educational institutions, teacher labor markets, and policy implementation across countries and regions may lead to different patterns. Therefore, caution is warranted when generalizing these findings beyond similar rural and policy settings.

## 6. Conclusions

As education serves as a key channel through which human capital is formed, understanding how teacher-related institutional support affects cognitive development is essential. Using longitudinal data from the CFPS (2012–2020) and a quasi-experimental design, this study provides evidence from a developing context on how large-scale improvements in teacher-related institutional conditions enhance cognitive outcomes in rural China.

The findings show that improved teacher support is consistently associated with higher levels of cognitive ability in rural students. The mechanism analyses suggest that the program may work by increasing teacher quantity and improving students’ satisfaction with their teachers. Furthermore, the benefits are more evident among girls and students from poor families. These compensatory developmental processes reflect the cognitive disadvantages associated with gender and poverty among these students.

These findings contribute to the human capital and educational inequality literature by showing that teacher-related institutional improvements meaningfully influence cognitive development in underserved rural areas. In practice, the results indicate that differentiated teacher-support strategies should not only address staff shortages but also strengthen teacher quality and improve the socio-emotional climate of classrooms. Specialized programs targeting schools with limited staffing, students from low-income families, and gender-sensitive educational needs may help promote more equitable developmental outcomes.

Future research could draw on richer and more representative data to examine additional pathways, such as classroom practices, pedagogical innovation, and motivational processes. It could also investigate longer-term outcomes, including non-cognitive skills and human capital trajectories.

This study contributes to the longitudinal evidence that strengthening teacher-related institutional support can improve children’s cognitive outcomes and reduce educational inequality. This study has implications not only for China but also for other developing contexts facing similar challenges.

## Figures and Tables

**Table 1:** Variable definitions and descriptive statistics.

Variables	Definitions	Observations	Mean	Min	Max	Standard Deviation
Total Cognitive Test Score	Sum of vocabulary and math calculation test scores, standardized annually	2996	−0.094	−4.421297	2.20251	0.9309502
Students’ Satisfaction with Teachers	1 if student’s satisfaction with homeroom teacher ≥3; 0 otherwise	2996	0.958	0	1	0.1996106
Teacher Quantity	Ratio of the total number of primary and secondary school teachers to the total population at year-end, expressed per 10,000 people	2996	86.831	18.2	215.3125	30.93745
RTSP Treatment Indicator	1 for treatment group, 0 for comparison group	2996	0.223	0	1	0.416091
Household Total Income	Sum of wage, business, property, transfer, and other income reported by all household members in the past year	2996	52,159.12	0	1,262,000	62,185.85
Housing Assets	Reported market value of the household’s housing properties	2996	414,750.28	0	50,200,000	1,176,709.93
Per Capita GDP	Regional Gross Domestic Product (GDP) divided by the total population at year-end	2996	33,625.769	4189.75	465,537.1	46,611.01
Proportion of Secondary Industry	Value of the secondary industry divided by regional GDP	2996	0.375	0.098689	0.699494	0.1640466
Proportion of Tertiary Industry	Value of the tertiary industry divided by regional GDP	2996	0.410	0.136025	0.715342	0.1308252
Per Capita Fixed-asset Investment	Total fixed-asset investment divided by the total population at year end	2996	2.751	0.027647	18.13303	1.910933
Gender	1 for male, 0 for female	2996	0.535	0	1	0.4988706
Age	/	2996	12.145	10	16	1.64251
Health Status	1 if health status is rated ≥4, otherwise 0	2996	0.968	0	1	0.1773296
Years of Education	/	2996	5.372	0	11	2.276477

**Table 2:** Baseline regression results.

	(1)	(2)
	Cognitive Test Total Score	Cognitive Test Total Score
Rural teacher support program	0.168 ***	0.185 ***
	(2.62)	(2.77)
Per capita GDP	−0.000000904	−0.00000166
	(−0.27)	(−0.49)
Proportion of secondary industry	0.0413	0.0578
	(0.13)	(0.18)
Proportion of tertiary industry	−0.146	−0.114
	(−0.29)	(−0.22)
Per capita fixed asset investment	0.0655 **	0.0705 **
	(1.96)	(2.03)
Gender	0.0469	0.0490
	(1.55)	(1.58)
Age	0.0468 **	0.0490 **
	(2.22)	(2.26)
Health status	0.0826	0.104
	(0.98)	(1.20)
Years of education	0.165 ***	0.162 ***
	(8.52)	(8.17)
Constant	−2.129 ***	−2.259 ***
	(−4.75)	(−4.89)
Family-level controls		Yes
County FE	Yes	Yes
Year FE	Yes	Yes
*N*	2996	2867
adj. *R*^2^	0.253	0.252

** *p* < 0.05, *** *p* < 0.01. Standard errors in parentheses. Columns (1) and (2) use the same outcome (cognitive test total score); Column (2) additionally includes family-level controls. Family-level controls include household total income and housing assets, both of which are log-transformed.

**Table 3:** Covariate balance before and after propensity score matching.

Covariates	Matching Status	Mean (Treated)	Mean (Control)	Std. Diff (%)	t-Value	*p*-Value
GDP per capita	Before matching	16,836	39,916	−73.2	−17.44	0.000
	After matching	20,018	20,259	−0.8	−0.64	0.521
Proportion of secondary industry	Before matching	0.26554	0.43257	−116.8	−30.29	0.000
	After matching	0.32138	0.32301	−1.1	−0.25	0.802
Proportion of tertiary industry	Before matching	0.46281	0.37842	68.9	18.05	0.000
	After matching	0.41829	0.41427	3.3	0.75	0.452
Per capita fixed-asset investment	Before matching	2.0105	2.9617	−60.2	−14.80	0.000
	After matching	1.9871	2.029	−2.7	−0.64	0.524
Gender	Before matching	0.57041	0.52081	10.0	2.64	0.008
	After matching	0.54172	0.57866	−7.4	−1.42	0.155
Age	Before matching	12.215	12.04	10.7	2.85	0.004
	After matching	12.216	12.337	−7.4	−1.38	0.167
Health status	Before matching	0.959	0.96692	−4.2	−1.12	0.261
	After matching	0.96306	0.95691	3.3	0.60	0.549
Years of education	Before matching	5.2816	5.3058	−1.1	−0.28	0.777
	After matching	5.342	5.4617	−5.3	−1.02	0.306

**Table 4:** Results of PSM-DID.

Variables	Cognitive Test Total Score
Rural teacher support program	0.299 **
	(0.117)
Constant	−1.015
	(0.814)
Control	Yes
County FE	Yes
Year FE	Yes
*N*	1063
adj. R^2^	0.253

** *p* < 0.05. Standard errors in parentheses. Control variables comprise the same set of individual-level, family-level, and time-varying county-level characteristics as in the baseline.

**Table 5:** Robustness test: accounting for overlapping policy effects.

Variables	Cognitive Test Total Score
Rural teacher support program	0.180 ***
	(2.76)
Constant	−2.234 ***
	(−5.90)
Control for national key poverty-stricken county program	Yes
County FE	Yes
Year FE	Yes
*N*	2996
adj. R^2^	0.255

*** *p* < 0.01. Standard errors in parentheses. All regressions include individual-, family-, and time-varying county-level characteristics as in the baseline specification, plus a policy variable constructed as the interaction between the national key poverty-stricken county indicator and year indicators to control for the concurrent poverty alleviation program.

**Table 6:** Mechanism test: teacher quantity.

	Teacher Quantity
Rural teacher support program	8.260 ***
	(11.14)
Per capita GDP	0.000529 ***
	(11.01)
Proportion of secondary industry	−14.76 ***
	(−4.18)
Proportion of tertiary industry	52.81 ***
	(8.90)
Per capita fixed-asset investment	−1.009 ***
Constant	41.25 ***
	(7.06)
Control	Yes
County FE	Yes
Year FE	Yes
*N*	396
adj. R^2^	0.958

*** *p* < 0.01. Standard errors in parentheses. Control variables comprise the same set of time-varying county-level characteristics as in the baseline.

**Table 7:** Mechanism test: students’ satisfaction with teachers.

	Students’ Satisfaction with Teachers
Rural teacher support program	0.0162 *
	(1.76)
Constant	1.182 ***
	(14.58)
Control	Yes
County FE	Yes
Year FE	Yes
*N*	2996
adj. R^2^	0.036

* *p* < 0.1, *** *p* < 0.01. Standard errors in parentheses. Control variables comprise the same set of individual-level, family-level, and time-varying county-level characteristics as in the baseline.

**Table 8:** Heterogeneity analysis by gender.

	Gender	
	(1)	(2)
	Boys	Girls
Rural teacher support program	0.0944	0.251 **
	(1.13)	(2.39)
Constant	−2.501 ***	−1.691 ***
	(−5.12)	(−3.69)
Control	Yes	Yes
County FE	Yes	Yes
Year FE	Yes	Yes
*N*	1616	1380
adj. R^2^	0.278	0.239

** *p* < 0.05, *** *p* < 0.01. Standard errors in parentheses. Control variables comprise the same set of individual-level (excluding gender), family-level, and time-varying county-level characteristics as in the baseline.

**Table 9:** Heterogeneity analysis by household income.

	Household Total Income	
	(1)	(2)
	High Income	Low Income
Rural teacher support program	0.0336	0.208 ***
	(0.23)	(2.80)
Constant	−0.142	−2.732 ***
	(−0.27)	(−6.46)
Control	Yes	Yes
County FE	Yes	Yes
Year FE	Yes	Yes
*N*	917	2079
adj. R^2^	0.271	0.252

*** *p* < 0.01. Standard errors in parentheses. Control variables comprise the same set of individual-level characteristics, housing assets, and time-varying county-level characteristics as in the baseline.

## Data Availability

The data for this study originate from the China Family Panel Studies (CFPS), available in both Chinese and English. CFPS provides comprehensive user guidelines and resources. Access to the CFPS data requires an application, as direct sharing or redistribution by individuals is not permitted. Detailed application information and video instructions are available upon account registration at https://cfpsdata.pku.edu.cn/#/home (accessed on 13 January 2026). Any questions regarding the data used in this study can be directed to the corresponding author.
